# Toxicological Safety, Antimicrobial Efficacy, and Sensory Evaluation of River Sand‐Derived Mica From Bangladesh: A Comprehensive Assessment for Cosmetic Applications

**DOI:** 10.1111/jocd.70448

**Published:** 2025-09-15

**Authors:** Hayatullah, Md. Golam Mostafa, Md. Aminur Rahman, Md. Nakib Hossen, Evena Parvin Lipy, Md. Ripaj Uddin, Mst. Maya Khatun, Md. Shazzadur Rahman

**Affiliations:** ^1^ Institute of Mining, Mineralogy and Metallurgy (IMMM) Bangladesh Council of Scientific and Industrial Research (BCSIR) Joypurhat Bangladesh; ^2^ Biomedical and Toxicological Research Institute (BTRI) Bangladesh Council of Scientific and Industrial Research (BCSIR) Dhaka Bangladesh; ^3^ Institute of National Analytical Research and Services (INARS) Bangladesh Council of Scientific and Industrial Research (BCSIR) Dhaka Bangladesh; ^4^ BCSIR Dhaka Laboratories Bangladesh Council of Scientific and Industrial Research (BCSIR) Dhaka Bangladesh

**Keywords:** cosmetics, health risk assessment, heavy metals, mica, skin irritation

## Abstract

**Background:**

Mica is widely applied in cosmetics, construction, automotive, electronics, defense, and medical industries, particularly important in cosmetics as an ingredient in foundations, eyeshadows, lipsticks, and nail polishes for its pearlescent and shimmering effects.

**Aims:**

To evaluate the suitability of mica minerals from Bangladeshi river sands for cosmetic applications by investigating their chemical composition, heavy metal content, potential for skin irritation, and antimicrobial activity.

**Methods:**

The chemical composition, crystallographic structure, and heavy metal content of mica samples were analyzed using X‐ray fluorescence, X‐ray diffraction, and Atomic Absorption Spectroscopy. Non‐carcinogenic and carcinogenic risks were evaluated using USEPA models (ADD, HQ, HI, LADD, ILTCR). Sensory evaluation was performed by 10 trained panelists, while primary skin irritation was tested on albino rabbits. Antimicrobial activity against bacterial and fungal strains was assessed using agar well diffusion and broth microdilution assays.

**Results:**

Muscovite contained high Al_2_O_3_ (30.46%) and SiO_2_ (49.29%), whereas biotite and phlogopite were enriched in Fe_2_O_3_ (43%) and MgO, supporting shimmer, color, and UV‐protective properties. XRD confirmed high crystallinity and purity. Heavy metals were within safe dermal and inhalation exposure limits. Sensory evaluation showed a silky texture, strong adhesion, and a luminous finish. All samples were non‐irritant (PII = 0.00). Biotite and phlogopite exhibited moderate antibacterial activity against *Staphylococcus aureus* , *Salmonella typhi* , and *Bacillus megaterium* , but no antifungal effects.

**Conclusions:**

Muscovite, biotite, and phlogopite mica exhibit low heavy metal content, non‐irritant and non‐carcinogenic behavior, moderate antibacterial activity, and favorable sensory properties, highlighting their potential as safe, effective, and multifunctional ingredients for cosmetic applications.

## 1 Introduction

Minerals have always played an important role in cosmetics, which have been a part of human culture for hundreds of years. Among these minerals, one that has found extensive use in cosmetics is mica, a naturally occurring hydrous potassium‐aluminum silicate mineral, owing to its specific mineralogical, crystallographic, and chemical properties [1]. These properties make mica a widely used ingredient in numerous cosmetic products like foundations, eyeshadows, lipsticks, and nail polishes [2]. This growth has to do mostly with increased consumer awareness and a heightened desire for cosmetic products, especially those derived from nature or minerals [3, 4].

Mica is used not only in cosmetic applications but also in a number of other industries, such as construction, automotive, electronics, defense, and medical devices. These properties are attributed to its high dielectric strength, thermal stability, and electrical resistance [5, 6]. Its significance in the cosmetic industry enhancing visual appearance/texture of cosmetic formulations [7] through its pearlescent/shimmering effect still remains unparalleled. In addition, various high‐performance formulations are turning more towards synthetic mica and applications like fluoro‐phlogopite (KMg_3_AlSi_3_O_10_F_2_) owing to its increased transparency and luster, making it ideal for use in products that can offer a natural, glowing appearance [8, 9].

In addition, with a change in consumer preference towards clean, eco‐friendly, and mineral cosmetics, there has been a high demand for naturally derived ingredients. The mineral cosmetics market held a value of 2.05 billion U.S. dollars worldwide in 2021, but it is expected to reach a value of 2.94 billion U.S. dollars by 2026, highlighting the increasing trend of incorporating minerals such as mica in cosmetic formulations [10]. Mica from the GMB river system: Mica is observed in heavy mineral deposits in Bangladesh in the GMB river system, which could act as a promising local deposit source of mica minerals for usage in the cosmetic industry.

Ironically, increased demand for mica has generated concerns over the ethical sourcing and safety of its use, despite its widespread application. Problems are emerging that draw increased scrutiny of the mica industry, such as child labor in the mining of mica, especially in nations such as India. In addition, concerns regarding heavy metal contamination and skin irritation potential of naturally sourced mica have raised demands for more stringent safety assessments [11, 12]. Therefore, it has now become important for the safety and viability of these mica minerals, especially supplied through the regions with less stringent regulations.

Based on these apprehensions, this study aims to characterize the mica minerals isolated from Bangladeshi river sand in terms of skin irritation potential, antimicrobial activity, and heavy metals content. This study aims to provide an evaluation of the local mica reserves and their potential use in cosmetics, assisting to better comprehend the safety of using such natural mica, efficacy, and the ethics of sourcing these minerals responsibly for cosmetics production. In the long run, it may establish a basis of increasing demand for eco‐friendly, nontoxic, and sustainable uses of cosmetic ingredients and will likewise assess the potential of untapped mineral reserves for the world outside the Bangladeshi cosmetic industry. To assess the sensory properties (texture, spreadability, adhesion, and visual appeal) of mica derived from river sand in Bangladesh for potential use in cosmetic formulations (e.g., eyeshadows, highlighters, foundations).

## 2 Materials and Methods

### 2.1 Sample Collection and Preparation

The Ganges‐Brahmaputra‐Meghna Delta is formed by the incoming major braided river system Brahmaputra River from the north. It contains large amounts of the mica minerals muscovite, biotite, and phlogopite. Industrial and cosmetic applications Recent studies indicate that these mica deposits are a naturally deposited, sustainable source for industrial and cosmetic applications.

**FIGURE 1:**
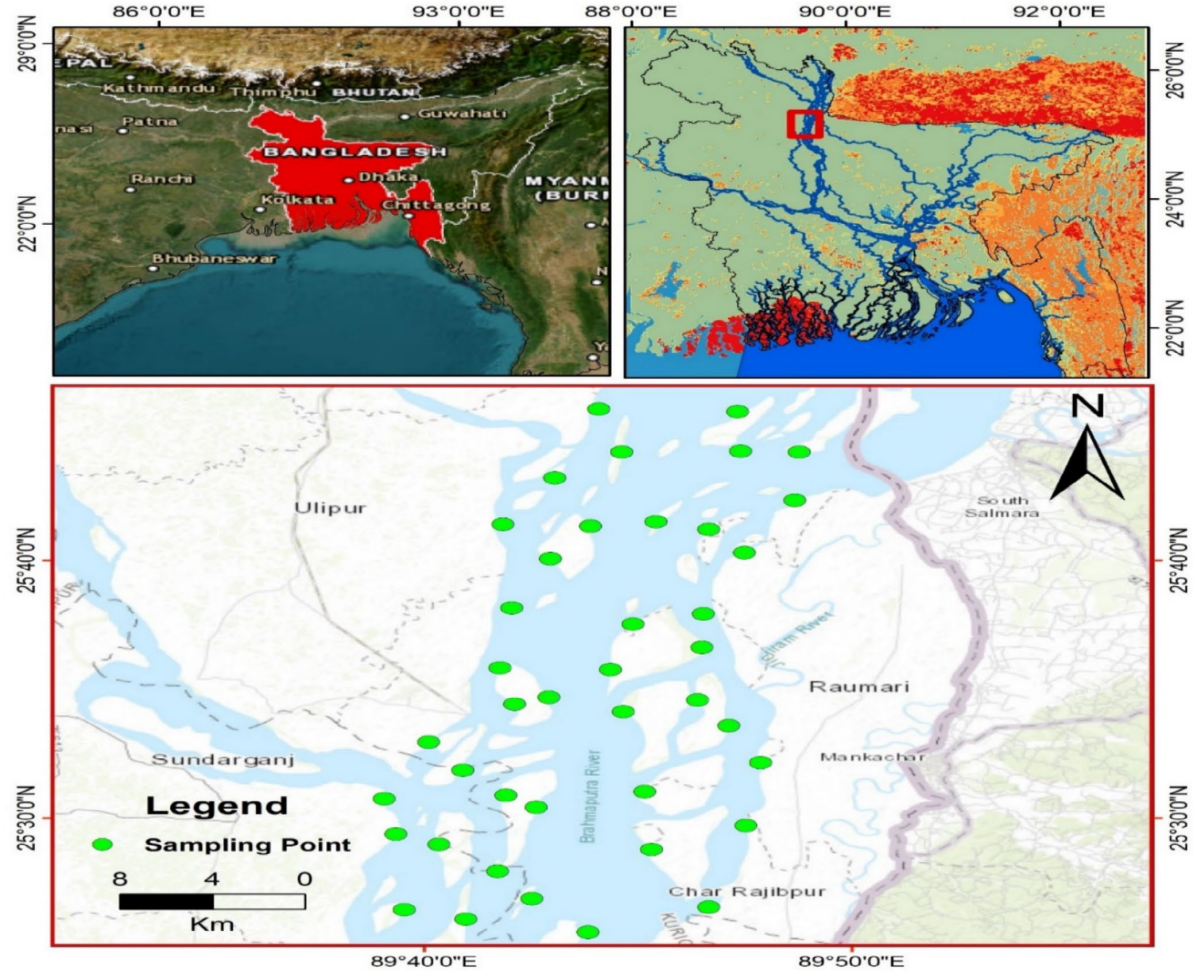
Location map of the study area.

The samples were collected (*n* = 40) from the Brahmaputra River that flows through the Gaibandha and Kurigram areas of Bangladesh (Figure 1). Air drying of the collected samples was done for 24 h before analysis. The heavy and light mineral fractions were separated from the samples by the shaking table. The dominant minerals in the heavy mineral (HM) fraction included magnetite, rutile, and zircon, whereas the light fraction (LF) was mostly composed of quartz and mica minerals.

**FIGURE 2:**
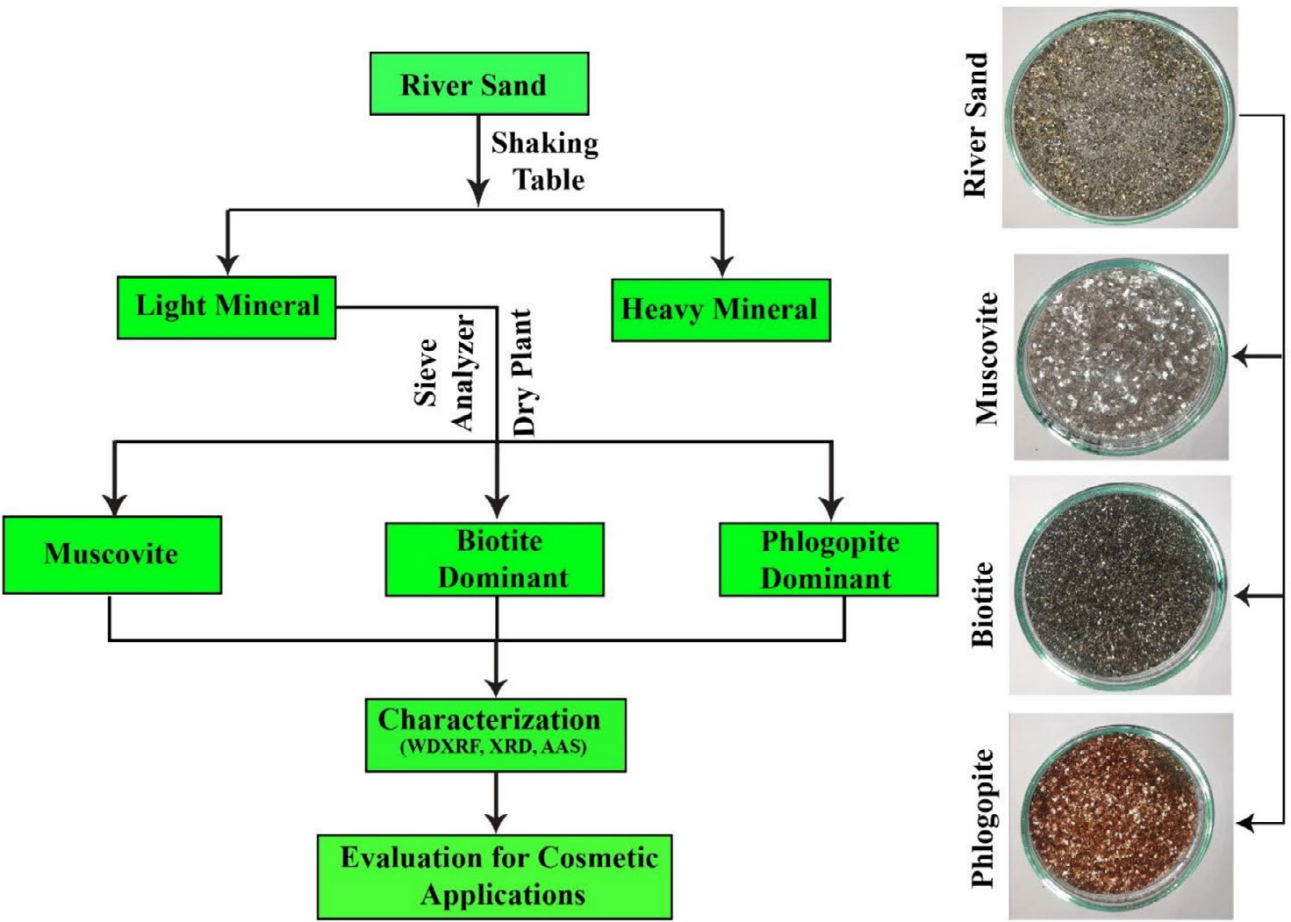
Flowchart of the preparation process for cosmetic products using mica.

The obtained light mineral was separated dry using Induced Roll Magnetic Separator (IRMS), Rare Earth Magnetic Drum Separator (REMDS), Electrostatic Plate Separator (EPS) and Rare Earth Magnetic Roll Separator (REMRS) methods. A sieve analyzer and hand magnet were then used to separate these minerals into relatively pure muscovite, biotite, and phlogopite minerals (Figure 2).

The purified mica was then treated using an attrition scrubber to remove adhering clay particles. Finally, full analyses of the purified mica samples were performed to establish the chemical composition and crystallographic structure, and to check for the presence of heavy metals. Muscovite, biotite, and phlogopite minerals were tested to determine their suitability for use in the cosmetics industry.

X‐ray fluorescence (XRF) spectroscopy (Rigaku ZSX Primus, 4 kW Rh‐anode X‐ray tube) was used to analyze the major and trace element content of the mica samples. Elemental profiling established the purity and quality of mica as a cosmetic grade raw material in the above way. The mineralogical and morphological properties of the mica samples were characterized by X‐ray diffraction (XRD) (Panalytical XPERTPRO, PW3040/60, scanning rate of 2.4° 2*θ*/min) to identify the crystal phases and types of mica such as muscovite and biotite.

### 2.2 Elemental Analysis of Heavy Metals

Mica samples were analyzed for the concentrations of cadmium (Cd), lead (Pb), chromium (Cr), nickel (Ni), arsenic (As), zinc (Zn), copper (Cu), and cobalt (Co)using Atomic Absorption Spectroscopy (AAS) (Varian, Australia) at the Institute of National Analytical Research and Service (INARS), BCSIR, Dhaka, Bangladesh.

For sample digestion, 10 g of sediment was weighed accurately into pre‐cleaned 250 mL glass beakers. Into each beaker was added a digestion mixture of 20 mL concentrated nitric acid (HNO_3_) and 10 mL perchloric acid (HClO_4_). Subsequently, the samples were dried on the hot plate at 150°C–180°C until complete organic matter evaporation. The analyzed residues were washed, filtered with Whatman qualitative filter paper No. 1, and diluted to 50 mL with nitric acid. The solutions were then re‐heated, concentrated, and transferred to 100 mL volumetric flasks, brought to the mark with deionized water (18.2 MΩ·cm), and filtered into acid‐cleaned polyethylene bottles for their analysis. Quality control was performed by processing blank samples at the same time following the method described by [13].

Analytical grade reagents were used throughout these experiments (Scharlau, Spain), and stringent controls were maintained on contamination (all glassware was cleansed with 10% nitric acid prior to analysis). For some components, specific equipment was applied, such as for cadmium analysis (Zeeman AAS) and arsenic quantification (SpectrAA AAS system).

Analytical accuracy and precision were ensured by the preparation of calibration curves that were generated with certified working standard solutions. All samples were measured in triplicate, and the accuracy was also confirmed by spike recovery tests, for which a range of 90%–110% accordance was obtained, and RSD was kept below 5%. Also, routine analysis of reagent blanks, calibration standards, and certified reference materials (CRM) (all purchased from Fluka, Germany, and traceable to NIST standard) was performed for quality assurance according to the protocol laid out by [14].

### 2.3 Risk Assessment of Non‐Carcinogenic Toxicity From Heavy Metal Exposure

The United States Environmental Protection Agency (USEPA) developed a model in 2001 for the evaluation of potential health risks, including heavy metal‐contaminated particulate matter (PM) exposure via oral ingestion, dermal contact, and inhalation pathways to assess the effects of such exposure on human health [15, 16]. This model evaluates health risks for children and adults and classifies them into carcinogenic effects and non‐carcinogenic effects. The non‐carcinogenic health risk was calculated using Equations ((1), (2), (3)). These equations compute risk using daily dose values, which are computed for each trace metal and for each route of exposure separately.

(1) $${\mathrm{ADD}}_{\mathrm{ing}}=\frac{C*\text{IngR}*\mathrm{ED}*\mathrm{EF}}{\mathrm{BW}*\mathrm{AT}}\times 10^{-6}$$

(2) $${\mathrm{ADD}}_{\mathrm{inh}}=\frac{C*\text{InhR}*\mathrm{ED}*\mathrm{EF}}{\mathrm{BW}*\mathrm{AT}*\mathrm{PEF}}$$

(3) $${\mathrm{ADD}}_{\mathrm{der}}=\frac{C*\mathrm{SA}*\mathrm{CF}*\mathrm{SL}*\mathrm{ABS}*\mathrm{ED}*\mathrm{EF}}{\mathrm{BW}*\mathrm{AT}}\times 10^{-6}$$

where ADD_ing_ = average daily dose caused by ingestion exposure (mg/kg/day); ADD_inh_ = average daily dose caused by inhalation exposure (mg/kg/day); ADD_der_ = average daily dose caused by dermal contact exposure (mg/kg/day); C is the contaminant concentration, IngR is the ingestion rate, InhR is the inhalation rate, SA is the skin surface area, CF is the conversion factor, SL is the powder adherence factor, ABS is the dermal absorption fraction, ED is the exposure duration, EF is the exposure frequency, BW is the body weight, AT is the averaging time, and PEF is the particle emission factor.

The hazard quotient (HQ) and hazard index (HI) are common tools for estimating the non‐cancer health risk of metal exposure. The HQ is the ratio of the estimated dose of a metal to which a subject is exposed (ADD) to the reference dose (RfD), that is, the maximum safe limit of exposure. The HQ values for different routes of exposure (ingestion (HQ_ing_), inhalation (HQ_inh_) and dermal contact (HQ_der_)) are calculated according to the following equations:

(4) $${\mathrm{HQ}}_{\mathrm{ing}}=\frac{A{\mathrm{DD}}_{\mathrm{ing}}}{\mathrm{RfD}}$$

(5) $${\mathrm{HQ}}_{\mathrm{inh}}=\frac{{\mathrm{ADD}}_{\mathrm{inh}}}{\mathrm{RfD}}$$

(6) $${\mathrm{HQ}}_{\mathrm{der}}=\frac{{\mathrm{ADD}}_{\mathrm{der}}}{\mathrm{RfD}}$$

The hazard index (HI) is then determined by summing the HQ values from all exposure routes, as shown in Equation (7):

(7) $$\mathrm{HI}=\sum {\mathrm{HQ}}_{\mathrm{ing}}+{\mathrm{HQ}}_{\mathrm{inh}}+{\mathrm{HQ}}_{\mathrm{der}}$$

HI values of ≤ 1 indicate no potential health risk associated with the exposure of metals. If the HE is > 1, it indicates that the combined exposure has the potential to exert adverse health effects [17, 18].

### 2.4 Assessment of Cancer Risk due to Heavy Metal Contamination

Using Equations (8) and (10), we calculated the cumulative average daily dose through ingestion, cutaneous exposure, and inhalation over a lifetime. The carcinogenic risk assessments associated with illegitimate exposure to heavy metal‐polluted mica powder were conducted by the ILTCR. USEPA recommendation acceptable cancer risks can generally be between 1 × 10^−6^ (i.e., 1 in 1 000 000) and 1 × 10^−4^ (i.e., 1 in 10 000); the ILTCR values for Pb, Cd, Cr, and As were calculated using Equations (12) and (13). The ILTCR of each heavy metal was calculated by the product of its Lifetime Average Daily Dose (LADD) for ingestion (LADD_ing_) and inhalation (LADD_inh_) with the corresponding Cancer Slope Factors (CSF_ing_ and CSF_inh_).

(8) $${\text{LADD}}_{\mathrm{ing}}=\frac{C*\mathrm{CF}*\mathrm{EF}}{\mathrm{AT}}\times \left(\frac{\text{IngR}\;\left(\text{child}\right)*\mathrm{ED}\;\left(\text{child}\right)}{\mathrm{BW}\;\left(\text{child}\right)}+\frac{\text{IngR}\;\left(\text{adult}\right)*\mathrm{ED}\;\left(\text{adult}\right)}{\mathrm{BW}\;\left(\text{adult}\right)}\right)$$

(9) $${\text{LADD}}_{\mathrm{inh}}=\frac{C*\mathrm{EF}}{\mathrm{PEF}*\mathrm{AT}}\times \left(\frac{\text{InhR}\;\left(\text{child}\right)*\mathrm{ED}\;\left(\text{child}\right)}{\mathrm{BW}\;\left(\text{child}\right)}+\frac{\text{InhR}\;\left(\text{adult}\right)*\mathrm{ED}\;\left(\text{adult}\right)}{\mathrm{BW}\;\left(\text{adult}\right)}\right)$$

(10) $${\text{LADD}}_{\mathrm{der}}=\frac{C*\mathrm{CF}*\mathrm{EF}*\mathrm{SL}*\mathrm{ABS}}{\mathrm{AT}}\times \left(\frac{\mathrm{SA}\;\left(\text{child}\right)*\mathrm{ED}\;\left(\text{child}\right)}{\mathrm{BW}\;\left(\text{child}\right)}+\frac{\mathrm{SA}\;\left(\text{adult}\right)*\mathrm{ED}\;\left(\text{adult}\right)}{\mathrm{BW}\;\left(\text{adult}\right)}\right)$$

(11) $$\mathrm{CR}={\text{LADD}}^{*}\mathrm{CSF}\;\left(\mathrm{CSF}=\text{Cancer slope factor}\right)$$

(12) $${\text{ILTCR}}_{\mathrm{inh}}={\text{LADD}}_{\mathrm{inh}}*{\mathrm{CSF}}_{\mathrm{inh}}$$

(13) $${\text{ILTCR}}_{\mathrm{ing}}={\text{LADD}}_{\mathrm{ing}}*{\mathrm{CSF}}_{\mathrm{ing}}$$

where LADD_inh_ = lifetime average daily dose caused by inhalation exposure (mg/kg/day); LADD_der_ = lifetime average daily dose caused by dermal contact exposure (mg/kg/day); LADD_ing_ = lifetime average daily dose caused by ingestion exposure (mg/kg/day); ILTCR = incremental life time cancer risk caused by inhalation exposure.

### 2.5 Sensory Test

The sensory evaluation follows a modified protocol based on ISO 24444:2019 (Cosmetics—Sun protection test methods—In vivo determination of the sun protection factor (SPF)) and ASTM E1490‐11 (Standard Guide for Sensory Evaluation of Products by Children and Adults) for panel testing [19]. ASTM E1490‐11 testing 10 trained panelists (cosmetic scientists, dermatologists, or experienced users) participated in the test. Participants have no known skin allergies to minerals or cosmetics [20]. Mica powder is blended into a base formulation (e.g., silicone‐based primer, pressed powder, or liquid foundation). A control sample (commercial‐grade mica) is used for comparison (Table 1).

### 2.6 Primary Skin Irritation Test

This method is designed to assess the potential health hazards associated with dermal exposure to a liquid or solid test substance. In accordance with the OECD‐recommended sequential testing strategy, the procedure begins with the performance of validated and accepted in vitro or ex vivo tests for corrosion or irritation.

In cases where in vivo testing is required, the albino rabbit is the preferred laboratory animal. Specifically, the test substance—either 0.5 mL for liquids or 0.5 g for solids—is applied in a single dose to a small shaved area of approximately 6 cm^2^ on the skin. Meanwhile, an untreated area on the same animal serves as the control. The exposure period lasts for 4 h, after which the residual test substance is thoroughly removed.

The method consists of two phases: an initial test followed by a confirmatory test, which is performed only if no corrosive effects are observed in the initial phase. Over the next 14 days, the animals are closely observed for signs of erythema (redness) and edema (swelling). Dermal irritation is evaluated based on the severity, nature, and reversibility of any lesions. If the reactions persist until the end of the 14‐day observation period, the test substance is classified as an irritant.

### 2.7 Antibacterial and Antifungal Activity Assays

The antibacterial and antifungal activities of the test substances were evaluated using four bacterial strains—*Escherichia coli, Staphylococcus aureus, Salmonella typhi*, and *Bacillus megaterium* —as well as two fungal species, *Aspergillus niger* and *Aspergillus flavus*. The agar well diffusion method (1) and broth microdilution method were employed to assess the inhibitory effects on both bacterial and fungal growth.

For bacterial assays, sterile Mueller–Hinton agar (Bio‐Rad, France) was used, while potato dextrose agar (Bio‐Rad, France) was utilized for fungal tests [21]. A fresh cell suspension was prepared for each microorganism: 0.1 mL of bacterial suspension, adjusted to 10^7^ CFU/mL, and 0.1 mL of fungal spore suspension, adjusted to 10^6^ spores/mL, were inoculated onto the respective agar plates. Wells (6 mm in diameter) were created in the agar using a sterile Pasteur pipette, and 50 μL of each sample solution was introduced into the wells. As a control, 50 μL of DMSO was used.

After allowing the plates to stand for 2 h to enable sample diffusion, they were incubated at 37°C for 24 h for bacterial cultures and at 28°C for 72 h for fungal cultures. All assays were performed in triplicate to ensure the reliability and reproducibility of the results.

### 2.8 Statistical Analysis

The data collected from storage materials and sensory analysis underwent statistical analysis were conducted using Lab Origin (Pro‐9). Significant differences between mean values were assessed using *p*‐values from two‐tailed *t*‐tests for three samples, and one‐way ANOVA (Analysis of Variance) for more than two samples, with a significance threshold set at *p* (0.05).

## 3 Result and Discussions

### 3.1 Chemical Compositions Analysis

Muscovite, phlogopite, and biotite micas display notable variations in their chemical compositions, which highlight differences in their geological formation environments and potential uses. Muscovite is characterized by high concentrations of Al_2_O_3_ (30.459%) and SiO_2_ (49.293%), along with a significant amount of K_2_O (11.3993%). These values are indicative of its origin in aluminum‐rich, iron‐poor geological settings, typical of potassium‐aluminum silicates. In contrast, both phlogopite and biotite contain markedly higher levels of Fe_2_O_3_ (approximately 43%) and MgO, measured at 5.7212% and 6.7012%, respectively (Table 2).

**TABLE 1:** Sensory testing parameters and scoring (5‐point scale) for mica.

Attribute	Evaluation criteria	Scale (1–5)
Texture	Grittiness, smoothness, and particle uniformity	1 (gritty) → 5 (silky)
Spreadability	Ease of blending on skin (fingertip or brush application).	1 (patchy) → 5 (even)
Adhesion	Longevity without fallout (tested under controlled humidity/temperature)	1 (poor) → 5 (excellent)
Visual finish	Pearlescence, opacity, and color payoff	1 (dull) → 5 (luminous)
Skin feel	Comfort after application (non‐drying, non‐irritating)	1 (irritating) → 5 (comfortable)

There are different types of micas used in certain cosmetic formulations such as muscovite, phlogopite, and biotite. With high SiO_2_ and Al_2_O_3_ content, muscovite provides a smooth‐to‐the‐touch and durable texture with a bit of reflectiveness to it, rendering it excellent for powders, foundations, and highlighters and trace elements such as ZnO and TiO_2_, which protect against ultraviolet rays, complementing its use for sun‐protective makeup. Unto phlogopite which has robust Fe_2_O_3_ and MgO contents provides a sparkling golden sheen and staying power time outperform for shadows, bronzers, and sunscreens to biotite which is high due to the same mineralization specifics so garnet can conceivably create intense, darker shades for bronzers, smokey eye shadows or tints while biotite on the other contains UV blocking properties as well as component stability enhancement (Table 2).

**TABLE 2:** Chemical composition (%) of muscovite, phlogopite, and biotite.

Chemical composition (%)	Muscovite	Phlogopite	Biotite
Na_2_O	1.0464	0.0384	0.0384
MgO	1.2812	5.7212	6.7012
Al_2_O_3_	30.459	17.092	18.292
SiO_2_	49.293	26.241	27.241
P_2_O_5_	0.0275	0.3961	0.3961
SO_3_	0.0116	0.0266	0.0266
Cl	0.0124	0.0394	0.0394
K_2_O	11.3993	3.7856	2.6856
CaO	0.3465	1.0065	1.1065
TiO_2_	1.1315	2.2801	2.1801
Fe_2_O_3_	4.5471	43.1009	43.2009
ZnO	0.0105	0.0774	0.0764
Ga_2_O_3_	0.0119	0.0132	0.0142
Rb_2_O	0.0832	0.1002	0.1012
ZrO_2_	0.051	0.0368	0.0378
Nb_2_O_5_	0.0073	0.0137	0.0127
BaO	0.2234	—	—
As_2_O_3_	—	0.0076	0.0066
V_2_O	0.0191	—	—
SrO	—	0.0086	0.0096
MnO	0.0381	—	—
CuO	—	0.0147	0.0137

Muscovite, phlogopite, and biotite have particular mineral compositions that facilitate texturing, impart sheen, and improve stability, making them staples in the development of new imaginative, performing cosmetics that respond to a host of aesthetic and functional requirements, from glimmering highlighters to long‐lasting foundations and makeup which protects from sunlight [22, 23].

### 3.2 X‐Ray Diffraction (XRD) Analysis

**FIGURE 3:**
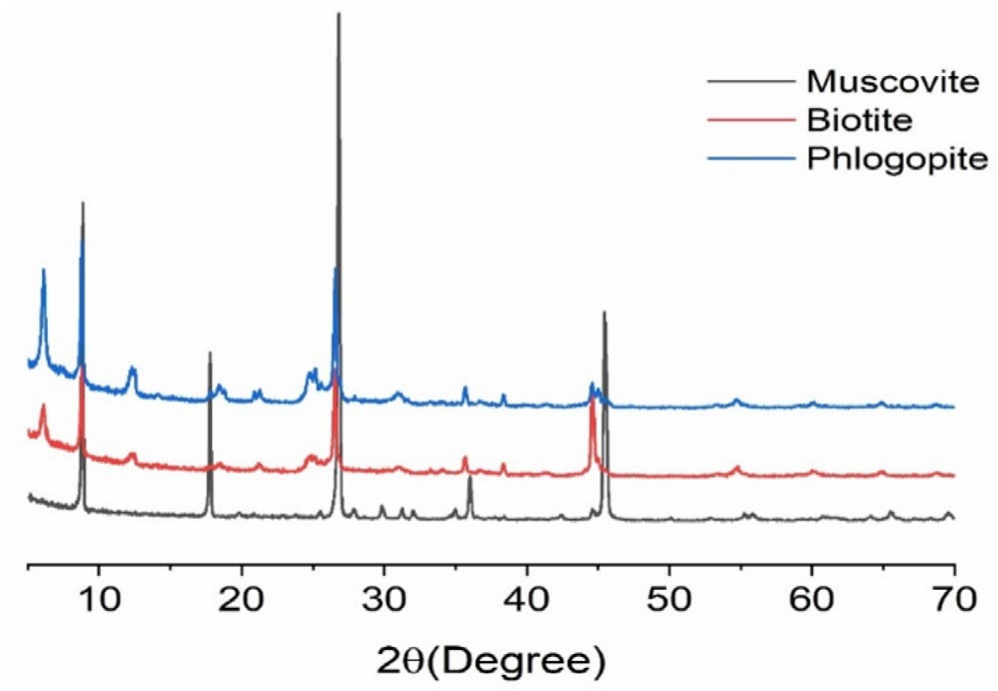
X‐ray diffraction (XRD) patterns of muscovite, biotite, and phlogopite.

X‐ray Diffraction (XRD) data on mica minerals such as muscovite, biotite, and phlogopite exhibit their layered silicate structures and elevated crystallinity, both essential for their functional applications across numerous industrial fields. Muscovite (KAl_2_(AlSi_3_O_10_)(OH)_2_) has strong and sharp basal reflections, 2*θ* values of 8.8°, 17.8°, and 26.6° corresponding to a basal spacing of 10 Å [24]. While the structures of biotite and phlogopite are similar, small differences in peak intensity and position are partially attributed to differences in their Fe‐Mg composition [25]. These structural dispositions correlate with the physical properties (e.g., luster, flexibility, and optical transparency) of the sheets that result in a high demand for mica flakes in the cosmetic industry [26]. The crystalline nature and purity of mica play a vital role in determining its usage in cosmetics, as evidenced by XRD (Figure 3).

## 4 Health Risk Assessment

### 4.1 The Non‐Carcinogenic Health Risk (NcHR)

#### 4.1.1 Hazard Quotient (HQ)

Using hazard quotient (HQ) values as indicators of potential exposure risks for non‐carcinogenic human health effects of heavy metals (Cr, As, Ni, Cu, Se, Cd, Pb) in children and adults, the study assessed human health risks for muscovite, biotite, and phlogopite.

The observed values of HQ_der_ for Cr, As, Ni, Cu, Se, Cd, and Pb metals for children and adults in muscovite, biotite, and phlogopite samples are shown in Table S1. The values of HQ obtained for all three samples were far below the threshold level of 1.0, which is taken as the acceptable limit for non‐carcinogenic effects [27]. Overall, the estimated risk values associated with dermal contact with these heavy metals indicate that there is no non‐carcinogenic risk of concern for both children and adults. Children's and adults' HQ_ing_ values representing ingestion exposure for the same metals were also observed for women and men. These HQ_ing_ values were also well below the indicated safety threshold, supporting the overall conclusion that exposure to these metals is not a non‐carcinogenic health risk. Additionally, the inhalation exposure risk expressed through preliminary valuations of HQ_inh_ was also computed in terms of the muscovite, biotite, and phlogopite samples studied for the metals of Cr, As, Ni, Cu, Se, Cd, and Pb, respectively. These values indicate that inhalation exposure to these metals does not represent a significant non‐carcinogenic risk in both groups. The classification of minimal dermal risk, as denoted by HQ < 1, but in line with those of the current study, suggests further support for a finding of minimal dermal risk. The lower HQ values observed in muscovite, biotite, and phlogopite study suggest that these products may have an even lower potential risk of developing ARI than those examined in previous studies. This study further demonstrates that, generally, at the levels of exposure experienced (based on typical product use patterns) heavy metals (such as Cr, As, Ni, Cu, Se, Cd and Pb) are not an immediate non‐carcinogenic risk, though higher concentrations will still be hazardous [28, 29]. In addition, the findings support the idea that the concentrations of these metals in the products tested in muscovite, biotite, and phlogopite are at a safe level for children and adults, consistent with earlier studies on the safe usage of these materials by the general population. The collapsed muscovite, biotite, and phlogopite samples analyzed appear not to represent a human health risk for dermal or inhalation exposure to the relevant heavy metals.

#### 4.1.2 Hazard Index (HI)

As shown in Table 3, calculated values for Hazard Index (HI) with respect to muscovite, biotite, and phlogopite were all less than 1, suggesting that there is a low probability of adverse health effects. These values were significantly lower than those in the literature, including the studies by [30]. HI values in this study were lower compared with those calculated in the above‐mentioned studies, which again confirms that the use of these samples in cosmetic products does not pose a risk of adverse health effects. Thus, according to the HI values calculated and their comparison to the previous studies, the exposure to these minerals from the cosmetic formulations is not likely to produce adverse health effects, confirming safety for the consumer.

**TABLE 3:** Non‐carcinogenic and carcinogenic health risks of studied mica for cosmetic applications.

	Non‐carcinogenic health risk (NcHR)			Carcinogenic health risk (CR)		
	Hazard index, HI = ∑ (HQ_ing_ + HQ_der_ + HQ_inh_)			ILCR = incremental lifetime carcinogenic risk		
	Muscovite	Biotite	Phlogopite	Muscovite	Biotite	Phlogopite
Cr						
Children	8.89E−07	4.459E−04	4.974E−04	1.642E−11	8.234E−09	1.837E−08
Adult	9.53E−08	4.778E−05	5.329E−05			
As						
Children	2.27E−05	5.067E−05	1.395E−04	1.256E−11	2.807E−11	5.152E−09
Adult	2.43E−06	5.429E−06	1.495E−05			
Ni						
Children	2.85E−07	5.188E−06	5.752E−06	1.192E−09	2.177E−08	1.416E−08
Adult	3.05E−08	5.559E−07	6.164E−07			
Cu						
Children	5.29E−06	8.161E−06	6.852E−06	—	—	—
Adult	5.68E−07	8.756E−07	7.351E−07			
Se						
Children	8.00E−09	5.334E−09	1.067E−08	—	—	—
Adult	8.57E−10	5.715E−10	1.143E−09			
Cd						
Children	2.67E−08	2.934E−07	5.067E−07	6.238E−13	6.862E−12	3.119E−11
Adult	2.86E−09	3.143E−08	5.490E−08			
Pb						
Children	1.62E−06	6.069E−06	8.823E−06	5.916E−12	2.223E−11	3.802E−09
Adult	1.73E−07	6.503E−07	9.460E−07			

### 4.2 Carcinogenic Risk (CR)

#### 4.2.1 Incremental Lifetime Cancer Risk (ILCR)

Incremental Lifetime Cancer Risk (ILCR) was calculated to evaluate the Incremental Lifetime Cancer Risk (ILCR) posed by the carcinogen elements such as Chromium (Cr), Arsenic (As), Nickel (Ni), Copper (Cu), Selenium (Se), Cadmium (Cd) and Lead (Pb) in muscovite, biotite, and phlogopite samples. Potential health risks from ingestion and inhalation pathways killed all age groups, including adults (adult group: EF‐adult, EF‐adult) and children (child group: EF‐kids, EF‐kids) sampling points, for which the results are summarized in Table 3. According to Li et al. [31], the carcinogenic risk (CR) is the lifetime probability that an individual will develop cancer given exposure to a carcinogenic substance. The CR in this study was determined by multiplying the LADD in mg kg^−1^ day^−1^, estimated according to Khairy et al. [32]. LADD values were determined for all sampling sites, indicating all impacted areas by anthropogenic activities that were used for risk assessment. In addition, Rahman et al. [18] stated that a cancer risk (CR) value between 1 × 10^−6^ and 1 × 10^−4^ is considered acceptable or tolerable. If CR > 1 × 10^−4^, it indicates an unacceptable risk, suggesting that the potential health impacts could be significant. Conversely, a CR value less than 1 × 10^−6^ is considered to pose no significant health threat. Based on the findings, all types of mica examined in this study are deemed suitable for use in the cosmetic industry.

CR values of Cr, As, Ni, Cu, Se, Cd, and Pb in muscovite, biotite, and phlogopite samples (as presented in Table 3) were less than 1 × 10^−6^. Confirming that the carcinogenic risk from these elements is very weak; therefore, the use of these samples for cosmetic purposes does not create any serious health hazards. The values are still within tolerable limits, indicating all the samples from the respective sites are safe for use, especially in beauty products where humans could be exposed. Li et al. [31] and Khairy et al. [32] showed similar results for other mineral samples regarding carcinogenic or potential carcinogenic risk. In addition, the low CR values obtained in this study confirm the effectiveness of natural mineral compositions for decreasing the carcinogenic risk, reinforcing the safe use of muscovite, biotite and phlogopite in cosmetic formulations.

The muscovite, biotite, and phlogopite had no health hazard, as reflected by Incremental Lifetime Cancer Risk (ILCR) for the elements Cr, As, Ni, Cu, Se, Cd, and Pb. Thus, having CR values below the aforementioned acceptable level of 1 × 10^−6^, these minerals are permitted in cosmetics as well, supporting their assertion for applicability in this segment. These analyses align with regulatory efforts in cosmetic safety to protect consumers by providing information for no‐effect levels of common cosmetic agents.

### 4.3 Status of Sensory Test

Ten panelists were assigned to evaluate the samples, with scores recorded in Table 4. Here, Codes A, B, and C represent river sand‐derived muscovite, phlogopite, and biotite, respectively, while Codes D, E, and F denote their commercial counterparts.

**TABLE 4:** Sensory evaluation of our developed mica and commercial mica.

Attribute	Code	Score out of 5.0	(%) CV	LSD value	Level of sign.	Remarks
Texture	A	4.0	0.047	0.024	*	Good
	B	3.95	0.047	0.024	*	Good
	C	3.95	0.047	0.025	*	Good
	D	4.0	0.065	0.032	*	Good
	E	4.0	0.065	0.032	*	Good
	F	4.0	0.065	0.032	*	Good
Spreadability	A	3.9	0.04	0.021	*	Good
	B	3.95	0.025	0.012	*	Good
	C	3.9	0.062	0.031	*	Good
	D	4.0	0.021	0.014	*	Good
	E	4.0	0.021	0.014	*	Good
	F	4.0	0.021	0.014	*	Good
Adhesion	A	3.95	0.047	0.024	*	Good
	B	4.0	0.047	0.024	*	Good
	C	3.95	0.047	0.029	*	Good
	D	4.0	0.065	0.031	*	Good
	E	4.0	0.065	0.030	*	Good
	F	4.0	0.065	0.030	*	Good
Visual finish	A	4.0	0.04	0.031	*	Good
	B	3.95	0.025	0.025	*	Good
	C	4.0	0.062	0.029	*	Good
	D	4.0	0.021	0.026	*	Good
	E	4.0	0.021	0.027	*	Good
	F	4.0	0.021	0.028	*	Good
Skin feel	A	4.0	0.047	0.030	*	Good
	B	4.0	0.047	0.032	*	Good
	C	3.5	0.047	0.026	*	Good
	D	4.0	0.065	0.031	*	Good
	E	4.0	0.065	0.032	*	Good
	F	4.0	0.065	0.030	*	Good

*Note:* The means with same superscripts within a column are not significantly different at *p* < 0.05.

The sensory evaluation of the samples collected from river sand (A–C) and commercial mica (D–F) showed good performance (> 3.5; *p* < 0.05) for all evaluated attributes with the best scores observed for attributes viz. texture (sensor 3.95–4.0), adhesion (3.95–4.0) and visual finish (3.95–4.0). Schist and muscovite (A) and phlogopite (B) on average produced better quality with a skin feel consistent with commercial samples (4.0), whereas biotite (C) had comparatively lower scores (3.5). The low coefficient of variation (CV: 0.021–0.065) and high LSD (*p* < 0.05) demonstrate the reliability and reproducibility of the findings, indicating that river sand mica can potentially serve as a sustainable alternative to commercial grades, with slight improvement desired in biotite's skin feel.

### 4.4 Evaluation of the Primary Skin Irritation Test Index

Muscovite, biotite, and phlogopite were applied occlusively for four hours to the bald skin of the tested animals, respectively. At 30 min following the removal of the test sample, no erythema or oedema was evident. Standard health markers (e.g., body weight, daily food/water intake) indicated overall health status (i.e., no apparent toxicity) entailing weight gain within the 14‐day observation period for all biotite treatments. The Primary Irritation Index (PII) was 0.00 (i.e., biotite, muscovite, and phlogopite respectively and can be categorized as Negligible (the animal model, rat)) (Table 5).

**TABLE 5:** Skin irritation test for muscovite, biotite and phlogopite.

Parameter	Name of supplied sample	Primary irritation index (PII)	PII value	Response category [33]
Acute dermal irritation	Biotite	0.00	0–0.4	Negligible
			0.5–1.9	Slight
			2–4.9	Moderate
			5–8	Severe
	Muscovite	0.00	0–0.4	Negligible
			0.5–1.9	Slight
			2–4.9	Moderate
			5–8	Severe
	Phlogopite	0.00	0–0.4	Negligible
			0.5–1.9	Slight
			2–4.9	Moderate
			5–8	Severe

In the PSIT test with muscovite, biotite and phlogopite mica minerals, no skin irritation was reported, so it can be considered safe for topical use in cosmetics. There was no erythema (redness) or oedema (swelling) observed after removal of the minerals from rat shaved skin after four hours of occlusive application, indicating virtually no irritation. Biotite continued to have no adverse effects and the treatments did not alter average body weight over the 14‐day observation period. For all three mica minerals, PII calculated to be equal to 0.00 so all mica minerals categorized as “Negligible” irritants as per standard classification [33]. These results are in agreement with other recent studies exploring the dermal safety of mica minerals. For example, a study muscovite, the biotite, and phlogopite minerals are noted for showing no significant irritation in primary skin irritation testing [3]. Moreover, Gomes et al. [4] supported these observations, revealing that diverse mica minerals, such as muscovite and phlogopite, were evaluated as non‐irritant in dermal safety assessments, affirming their usefulness for sensitive skin. Thus, these findings substantiate the growing application of mica minerals in cosmetic products, in which safety and skin compatibility are of utmost importance.

### 4.5 Antimicrobial Activity

Biotite/phlogopite and muscovite in DMSO (1/3 (v/v)) extracts showed relevant amounts of antibacterial capacity. At an average of 9.0 to 12.0 mm inhibition zone, both samples completely inhibited the growth of *S. aureus, S. typhi*, and *B. megaterium* . The biotite/phlogopite sample had the strongest antibacterial activity against *S. aureus* , with the largest inhibition zone (12 mm) (Table 6). Meanwhile, the muscovite sample was moderately active against *B. megaterium* , with an inhibition zone of 11 mm, although the biotite/phlogopite sample exhibited more meager inhibition with an inhibition zone of 9 mm. They showed activity against *S. typhi* ; inhibition zone of biotite/phlogopite around 8 mm. Interestingly, neither sample was active against *E. coli* . Here, kanamycin (30 μg) was used as a positive control. For antifungal activity, neither sample was effective against *A. niger* or *A. flavus* (the standard antifungal agent was Ketoconazole [30 μg]). These results correlate well with Nahar et al. [34] which stated that the most antimicrobial activity of biotite on *Listeria monocytogenes, S. aureus, Salmonella abony, E. coli*, and *Candida albicans* . Furthermore, Catalina Haidău et al. [35] found that muscovite had little antibacterial inhibition against *B. megaterium* , which is consistent with the data observed in this study.

**TABLE 6:** Zone of inhibition (mm) of biotite/phlogopite and muscovite against various bacterial strains.

Samples	Zone of inhibition (mm)			
	*Escherichia coli*	*Staphylococcus aureus*	*Salmonella typhi*	*Bacillus megaterium*
Biotite/Phlogopite	—	12	8	9
Muscovite	—	—	—	11
Kanamycin (30 μg) (std)	21	22	22	21

**FIGURE 4:**
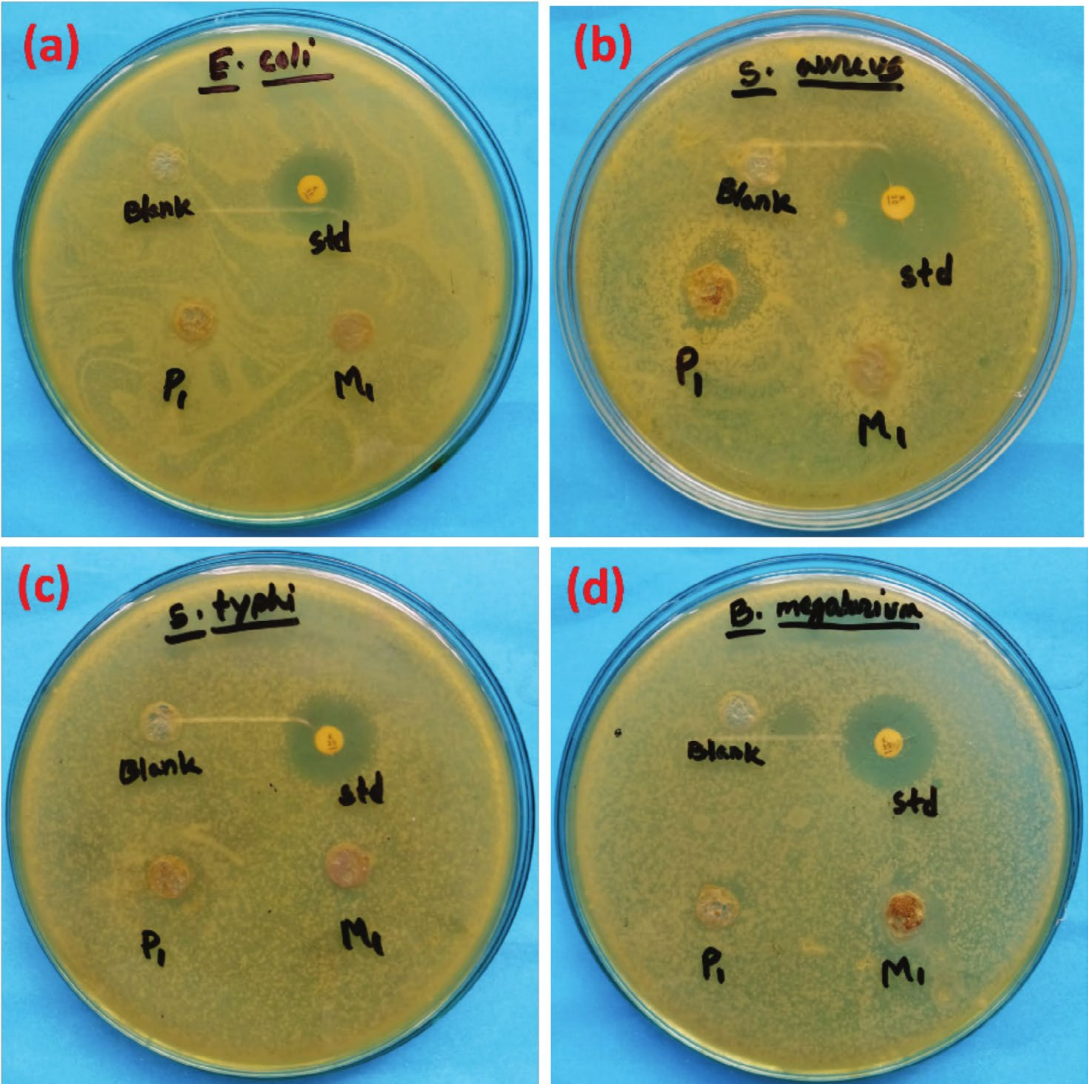
Antimicrobial activity of muscovite, biotite, and phlogopite against (a) *Escherichia coli* , (b) *Staphylococcus aureus* , (c) *Salmonella typhi* , and (d) *Bacillus megaterium*.

Muscovite and biotite/phlogopite mica samples were tested for antimicrobial activity using the agar well diffusion method (dissolved in DMSO at a dilution of 1/3 (v/v)). The antibacterial tests showed significative potency against *S. aureus, S. typhi*, and *B. megaterium* (inhibition zones between 8.0 and 12.0 mm), among several other pathogenic strains. The obtained most remarkable antibacterial activity in in vitro screening, with zones of inhibition reaching 12 mm against *S. aureus* which is a widespread skin pathogen and a suitable target for antimicrobial cosmetics was obtained for; biotite/phlogopite. Good antibacterial activity was also detected against *B. megaterium* , muscovite produced an inhibition zone of 11 mm, biotite/phlogopite = 9 mm. The biotite/phlogopite had a mild activity against *S. typhi* (8 mm inhibition zone) (Figure 4). While our data did not demonstrate activity against *E. coli* , use of both mica samples showed no inhibition of growth, highlighting the selectivity of this antibacterial potential.

As a positive control, kanamycin (30 μg) was used, and the inhibition zones (21–22 mm) were considerably larger, which indicates correct assay validity. In contrast, the antifungal assessment demonstrated no activity of either mica sample against *A. niger* or *A. flavus* (Ketoconazole (30 μg) as a standard antifungal reference). Biotite was found to have a similar strong antimicrobial effect against *S. aureus, L. monocytogenes, S. abony, and C. albicans*, but had a limited effect on *E. coli* . Similarly, Zada et al. [36], which showed minimal antibacterial activity of muscovite, particularly against *B. megaterium* , which can be seen with moderate inhibition zones in this study. The different degree of selective antimicrobial activity highlighted in the present study suggests that the effectiveness of mica minerals is determined not only by their chemical composition but also by the structural properties of the microbial cell wall, providing useful elements for the formulation of antibacterial cosmetics for gram‐positive bacteria.

## 5 Conclusions

The chemical composition, crystallographic structure, toxicological safety, and antimicrobial activity of muscovite, biotites, and phlogopite mica from Bangladeshi river sand have been comprehensively evaluated to provide insight into their potential applications in cosmetic formulations. The chemical compositions showed high purity of muscovite (high SiO_2_ and Al_2_O_3_), and biotite and phlogopite (high Fe_2_O_3_ and MgO), attributed to the diversified functions of these minerals in improving the texture, color, and stability of cosmetic products. This suggested their optical and mechanical characteristics are beneficial for stable and effective formulation behavior and, importantly, XRD analysis confirmed well‐defined crystalline structures.

As per toxicological evaluations, heavy metal levels in all samples were lower than internationally agreed thresholds for toxicity, and both non‐carcinogenic (HQ, HI < 1) and carcinogenic (ILCR < 1 × 10^−6^) indices confirmed their safety for adults and children. Moreover, primary skin irritation tests assigned all micas a non‐irritant classification, while biotite and phlogopite displayed moderate antibacterial activity against *S. aureus* and *B. megaterium* , that could be translated into formulations pertaining to antimicrobials claims. However, no antifungal activity was observed. The mica‐based formulations exhibited favorable sensory performance, with average scores ranging from 4.2 to 4.8 across evaluated attributes: texture (4.5), spreadability (4.6), adhesion (4.4), visual finish (4.8), and skin feel (4.7) highlighting their potential suitability for cosmetic applications. These results highlight muscovite, biotite, and phlogopite mica minerals as innovative, safe, and effective cosmetic ingredients that may provide such properties as including non‐irritant properties, aesthetic capacity, and antimicrobial action.

## Author Contributions

Hayatullah and Md. Golam Mostafa performed, designed, analyzed the data, and wrote the paper. Md. Aminur Rahman contributed essential reagents or tools. Md. Nakib Hossen, Evena Parvin Lipy, Md. Ripaj Uddin, Mst. Maya Khatun, and Md. Shazzadur Rahman analyzed the data.

## Ethics Statement

The study protocol, including the primary skin irritation test on animal models, was reviewed and approved by the Ethical Committee of BCSIR for Animal Research (ECAR), in accordance with national and institutional guidelines for the care and use of laboratory animals. The ethical approval reference number is 39.02.0000.088.99.002.23.ECAR.2025.18.

## Consent

All authors have read the final manuscript and approved it for publication.

## Conflicts of Interest

The authors declare no conflicts of interest.

## Supporting information


**Table S1:** Non‐carcinogenic and carcinogenic health risk assessment of studied mica (a. muscovite, b. biotite, c. phlogopite) sample from river sand, Bangladesh.

## Data Availability

All data are available within the manuscript.

## References

[1] J. D. D. Moraes , S. R. A. Bertolino , S. L. Cuffini , D. F. Ducart , P. E. Bretzke , and G. R. Leonardi , “Clay Minerals: Properties and Applications to Dermocosmetic Products and Perspectives of Natural Raw Materials for Therapeutic Purposes—A Review,” International Journal of Pharmaceutics 534, no. 1–2 (2017): 213–219, 10.1016/j.ijpharm.2017.10.031. 29038067

[2] F. D. Sarruf , V. J. P. Contreras , R. M. Martinez , M. V. R. Velasco , and A. R. Baby , “The Scenario of Clays and Clay Minerals Use in Cosmetics/Dermocosmetics,” Cosmetics 11, no. 1 (2024): 7, 10.3390/cosmetics11010007.

[3] H. Thakur , S. Kaur , A. Singh , G. B. Singh , and G. Mudgal , “Smart Sustainable Materials: A Blueprint for a Better Tomorrow,” in Functionalized Cellulose Materials, ed. K. K. Kesari , C. Prakash , M. Khalid , and A. Negi , in Engineering Materials (Springer Nature Switzerland, 2025), 253–276, 10.1007/978-3-031-76953-5_11.

[4] C. S. F. Gomes , D. F. G. Santos , and M. H. R. Amaral , “Minerals in Pharmacy and Cosmetics,” in Minerals Latu Sensu and Human Health, ed. C. Gomes and M. Rautureau (Springer International Publishing, 2021), 405–441, 10.1007/978-3-030-65706-2_9.

[5] P. Tenoriocavalcante , M. Dondi , G. Guarini , F. Barros , and A. Benvindodaluz , “Ceramic Application of Mica Titania Pearlescent Pigments,” Dyes and Pigments 74, no. 1 (2007): 1–8, 10.1016/j.dyepig.2006.01.026.

[6] M. Hosseini‐Zori , “Synthesis and Characterization of Red Pearlescent Pigments Based on Muscovite and Zirconia‐Nanoencapsulated Hematite,” Progress in Color, Colorants and Coatings (PCCC) 5 (2012): 7–13.

[7] T. Junru , H. Yunfang , H. Wenxiang , C. Xiuzeng , and F. Xiansong , “The Preparation and Characteristics of Cobalt Blue Mica Coated Titania Pearlescent Pigment,” Dyes and Pigments 52, no. 3 (2002): 215–222, 10.1016/S0143-7208(01)00086-9.

[8] V. Stengl , “The Preparation and Characteristics of Pigments Based on Mica Coated With Metal Oxides,” Dyes and Pigments 58, no. 3 (2003): 239–244, 10.1016/S0143-7208(03)00086-X.

[9] Q. Gao , X. Wu , Y. Fan , and X. Zhou , “Low Temperature Synthesis and Characterization of Rutile TiO_2_‐Coated Mica–Titania Pigments,” Dyes and Pigments 95, no. 3 (2012): 534–539, 10.1016/j.dyepig.2012.06.006.

[10] E. Wargala , M. Sławska , A. Zalewska , and M. Toporowska , “Health Effects of Dyes, Minerals, and Vitamins Used in Cosmetics,” Women 1, no. 4 (2021): 223–237, 10.3390/women1040020.

[11] N. Amberg and C. Fogarassy , “Green Consumer Behavior in the Cosmetics Market,” Resources 8, no. 3 (2019): 137, 10.3390/resources8030137.

[12] C. Faria‐Silva , A. Ascenso , A. M. Costa , et al., “Feeding the Skin: A New Trend in Food and Cosmetics Convergence,” Trends in Food Science and Technology 95 (2020): 21–32, 10.1016/j.tifs.2019.11.015.

[13] A. B. Hasan , A. H. M. S. Reza , M. A. B. Siddique , et al., “Origin, Spatial Distribution, Sediment Contamination, Ecological and Health Risk Evaluation of Trace Metals in Sediments of Ship Breaking Area of Bangladesh,” Journal of Hazardous Materials 465 (2024): 133214, 10.1016/j.jhazmat.2023.133214. 38101007

[14] N. A. Siddiquee , S. Parween , M. M. A. Quddus , and P. Barua , “Heavy Metal Pollution in Sediments at Ship Breaking Area of Bangladesh,” in Coastal Environments: Focus on Asian Regions, ed. V. Subramanian (Springer Netherlands, 2012), 78–87, 10.1007/978-90-481-3002-3_6.

[15] S. K. Frimpong and S. S. Koranteng , “Levels and Human Health Risk Assessment of Heavy Metals in Surface Soil of Public Parks in Southern Ghana,” Environmental Monitoring and Assessment 191, no. 9 (2019): 588, 10.1007/s10661-019-7745-0. 31444583

[16] N. Zheng , Q. Wang , X. Zhang , D. Zheng , Z. Zhang , and S. Zhang , “Population Health Risk due to Dietary Intake of Heavy Metals in the Industrial Area of Huludao City, China,” Science of the Total Environment 387, no. 1–3 (2007): 96–104, 10.1016/j.scitotenv.2007.07.044. 17765948

[17] A. S. S. Ahmed , M. B. Hossain , S. M. O. F. Babu , M. M. Rahman , and M. S. I. Sarker , “Human Health Risk Assessment of Heavy Metals in Water From the Subtropical River, Gomti, Bangladesh,” Environmental Nanotechnology, Monitoring & Management 15 (2021): 100416, 10.1016/j.enmm.2020.100416.

[18] M. S. Rahman , A. H. M. S. Reza , G. S. Sattar , et al., “Mobilization Mechanisms and Spatial Distribution of Arsenic in Groundwater of Western Bangladesh: Evaluating Water Quality and Health Risk Using EWQI and Monte Carlo Simulation,” Chemosphere 366 (2024): 143453, 10.1016/j.chemosphere.2024.143453. 39362382

[19] M. Pissavini , C. Tricaud , G. Wiener , et al., “Validation of a New *in Vitro* Sun Protection Factor Method to Include a Wide Range of Sunscreen Product Emulsion Types,” International Journal of Cosmetic Science 42, no. 5 (2020): 421–428, 10.1111/ics.12625. 32390187 PMC8246923

[20] T. F. Tadros , “Future Developments in Cosmetic Formulations,” International Journal of Cosmetic Science 14, no. 3 (1992): 93–111, 10.1111/j.1467-2494.1992.tb00045.x. 19272094

[21] S. Aliou , B. Fanou , J. R. Klotoé , et al., “Antimicrobial and Enzymatic Potential of *Pterocarpus erinaceus* Poir. Endophytes Used in Benin (West Africa),” Bulletin of the National Research Centre 48, no. 1 (2024): 128, 10.1186/s42269-024-01284-1.

[22] K. S. Randhawa , “Synthesis, Properties, and Environmental Impact of Hybrid Pigments,” Scientific World Journal 2024, no. 1 (2024): 2773950, 10.1155/tswj/2773950.

[23] T. I. Yushina , A. M. Dumov , N. Van Chon , N. T. Thuy , National University of Science and Technology—MISIS, Moscow, Russia , et al., “Mineral Composition and Commercial Application Feasibility of Sericite Ore in Ha Tinh Province,” Eurasian Mining 2 (2020): 32–38, 10.17580/em.2020.02.08.

[24] M. Vangu Seke , J.‐P. Mbo Nzundu , D. abitha Sola , et al., “Chemical, Mineralogical and Microbiological Characterization of Some Geophagic Clays From The Democratic Republic of the Congo: CBRN Risks,” Journal Africain Des Sciences 1, no. 2 (2024): 96–104, 10.70237/jafrisci.2024.v1.i2.11.

[25] R. Yang , Y. Fan , R. Ye , et al., “MnO_2_‐Based Materials for Environmental Applications,” Advanced Materials 33, no. 9 (2021): 2004862, 10.1002/adma.202004862. 33448089

[26] T. Deng , J. Li , G. Zhao , et al., “Visible‐Light Photoelectric Performance and Bending Stability of Flexible LaCoO_3_/Mica Thin Films,” Ceramics International 50, no. 15 (2024): 27165–27175, 10.1016/j.ceramint.2024.05.014.

[27] J. Ling , Z. Yan , X. Liu , et al., “Health Risk Assessment and Development of Human Health Ambient Water Quality Criteria for PCBs in Taihu Basin, China,” Science of the Total Environment 920 (2024): 170669, 10.1016/j.scitotenv.2024.170669. 38316297

[28] A. Sarker , J. E. Kim , A. R. M. T. Islam , et al., “Heavy Metals Contamination and Associated Health Risks in Food Webs—A Review Focuses on Food Safety and Environmental Sustainability in Bangladesh,” Environmental Science and Pollution Research 29, no. 3 (2022): 3230–3245, 10.1007/s11356-021-17153-7. 34739668 PMC8569293

[29] R. Nag , S. M. O'Rourke , and E. Cummins , “Risk Factors and Assessment Strategies for the Evaluation of Human or Environmental Risk From Metal(Loid)s—A Focus on Ireland,” Science of the Total Environment 802 (2022): 149839, 10.1016/j.scitotenv.2021.149839. 34455276

[30] H. Arshad , M. Z. Mehmood , M. H. Shah , and A. M. Abbasi , “Evaluation of Heavy Metals in Cosmetic Products and Their Health Risk Assessment,” Saudi Pharmaceutical Journal 28, no. 7 (2020): 779–790, 10.1016/j.jsps.2020.05.006. 32647479 PMC7335825

[31] Z. Li , Z. Ma , T. J. Van Der Kuijp , Z. Yuan , and L. Huang , “A Review of Soil Heavy Metal Pollution From Mines in China: Pollution and Health Risk Assessment,” Science of the Total Environment 468 (2014): 843–853, 10.1016/j.scitotenv.2013.08.090. 24076505

[32] M. A. Khairy , A. O. Barakat , A. R. Mostafa , and T. L. Wade , “Multielement Determination by Flame Atomic Absorption of Road Dust Samples in Delta Region, Egypt,” Microchemical Journal 97, no. 2 (2011): 234–242, 10.1016/j.microc.2010.09.012.

[33] M. Hemmati , A. Ghasemzadeh , M. H. Malek‐kheili , K. Khoshnevisan , and M. K. Koohi , “Investigation of Acute Dermal Irritation/Corrosion, Acute Inhalation Toxicity and Cytotoxicity Tests for Nanobiocide®,” Nanomedicine Research Journal 1 (2016): 23–29.

[34] A. Nahar , M. Sahadat Hossain , M. A. Akbor , et al., “Evaluation of Anti‐Microbial Activity of Biotite and Biotite Composite Based on Crystallographic Parameters: Estimation of Crystallite Size Employing X‐Ray Diffraction Data,” Results in Engineering 23 (2024): 102595, 10.1016/j.rineng.2024.102595.

[35] C. Haidău , R. Năstase‐Bucur , P. Bulzu , et al., “A 16S rRNA Gene‐Based Metabarcoding of Phosphate‐Rich Deposits in Muierilor Cave, South‐Western Carpathians,” Frontiers in Microbiology 13 (2022): 877481, 10.3389/fmicb.2022.877481. 35663904 PMC9161362

[36] S. Zada , A. A. Naseem , S.‐J. Lee , et al., “Geochemical and Mineralogical Analysis of Kashmir Cave (SMAST), Buner, Pakistan, and Isolation and Characterization of Bacteria Having Antibacterial Activity,” Journal of Cave and Karst Studies 78, no. 2 (2016): 94–109, 10.4311/2014MB0110.

