# Supplementary material for: Toxicological Safety, Antimicrobial Efficacy, and Sensory Evaluation of River Sand‐Derived Mica From Bangladesh: A Comprehensive Assessment for Cosmetic Applications
Source: J Cosmet Dermatol. 2025 Sep 15;24(9):e70448. doi: 10.1111/jocd.70448 (PMC12435160; doi:10.1111/jocd.70448)
Supplement: Supplementary file 1 — Table S1: Non‐carcinogenic and carcinogenic health risk assessment of studied mica (a. muscovite, b. biotite, c. phlogopite) sample from river sand, Bangladesh. [file JOCD-24-e70448-s001.docx]

**Toxicological and Antimicrobial Evaluation of Mica Minerals from River Sand in Bangladesh: A Comprehensive Study for Cosmetic Applications**

**Supplementary Information:**

**Table S1.** Non-Carcinogenic and Carcinogenic Health Risk assessment of studied Mica (a. Muscovite, b. Biotite, c. Phlogopite) sample from river sand, Bangladesh.

1. **Muscovite**

|  | | Non-Carcinogenic Health Risk | | | | |  | | Carcinogenic Health Risk | | | | |
| --- | --- | --- | --- | --- | --- | --- | --- | --- | --- | --- | --- | --- | --- |
|  |  | HQ_ing_ | HQ_inh_ | HQ_der_ | HI=∑HQ |  | | LADD_ing_ | | LADD_inh_ | LADD_der_ | ILCR |  |
| Cr | Children | 8.889E-07 | 2.484E-11 | 2.489E-15 | 8.889E-07 |  | | 3.265E-11 | | 2.079E-15 | 1.757E-13 | 1.642E-11 |  |
|  | Adult | 9.524E-08 | 1.401E-11 | 3.800E-16 | 9.525E-08 |  | |  |  |  |  |  |  |
| As | Children | 2.267E-05 | 6.333E-10 | 6.347E-14 | 2.268E-05 |  | | 8.327E-10 | | 5.302E-14 | 4.479E-12 | 1.256E-11 |  |
|  | Adult | 2.428E-06 | 3.571E-10 | 9.690E-15 | 2.429E-06 |  | |  |  |  |  |  |  |
| Ni | Children | 2.847E-07 | 7.954E-12 | 7.971E-16 | 2.847E-07 |  | | 6.972E-10 | | 4.439E-14 | 3.751E-12 | 1.192E-09 |  |
|  | Adult | 3.050E-08 | 4.485E-12 | 1.216E-16 | 3.051E-08 |  | |  |  |  |  |  |  |
| Cu | Children | 5.294E-06 | 1.479E-10 | 1.483E-14 | 5.294E-06 |  | | 1.651E-12 | | 1.395E-10 | 2.607E-08 | - |  |
|  | Adult | 5.672E-07 | 8.341E-10 | 2.263E-15 | 5.680E-07 |  | |  |  |  |  |  |  |
| Se | Children | 8.000E-09 | 2.235E-13 | 2.240E-17 | 8.001E-09 |  | | 4.898E-12 | | 3.119E-16 | 2.635E-14 | - |  |
|  | Adult | 8.571E-10 | 1.261E-13 | 3.420E-18 | 8.571E-10 |  | |  |  |  |  |  |  |
| Cd | Children | 2.667E-08 | 7.451E-13 | 7.467E-17 | 2.667E-08 |  | | 1.633E-12 | | 1.039E-16 | 8.784E-15 | 6.238E-13 |  |
|  | Adult | 2.857E-09 | 4.202E-13 | 1.140E-17 | 2.858E-09 |  | |  |  |  |  |  |  |
| Pb | Children | 1.615E-06 | 4.513E-11 | 4.240E-15 | 1.615E-06 |  | | 6.923E-10 | | 4.408E-14 | 3.724E-12 | 5.916E-12 |  |
|  | Adult | 1.731E-07 | 2.545E-11 | 6.905E-16 | 1.731E-07 |  | |  |  |  |  |  |  |

1. **Biotite**

|  | | Non-Carcinogenic Health Risk | | | | |  | | Carcinogenic Health Risk | | | | |
| --- | --- | --- | --- | --- | --- | --- | --- | --- | --- | --- | --- | --- | --- |
|  |  | HQ_ing_ | HQ_inh_ | HQ_der_ | HI=∑HQ |  | | LADD_ing_ | | LADD_inh_ | LADD_der_ | ILCR |  |
| Cr | Children | 4.459E-04 | 1.246E-08 | 1.249E-12 | 4.459E-04 |  |  | 1.638E-08 | | 1.043E-12 | 8.813E-11 | 8.234E-09 |  |
|  | Adult | 4.778E-05 | 7.026E-09 | 1.906E-13 | 4.778E-05 |  |  |  |  |  |  |  |  |
| As | Children | 5.067E-05 | 1.416E-09 | 1.418E-13 | 5.067E-05 |  |  | 1.861E-09 | | 1.185E-13 | 1.002E-11 | 2.807E-11 |  |
|  | Adult | 5.428E-06 | 7.983E-10 | 2.166E-14 | 5.429E-06 |  |  |  |  |  |  |  |  |
| Ni | Children | 5.188E-06 | 1.449E-10 | 1.453E-14 | 5.188E-06 |  |  | 1.271E-08 | | 8.093E-13 | 6.835E-11 | 2.177E-08 |  |
|  | Adult | 5.558E-07 | 8.174E-11 | 2.218E-15 | 5.559E-07 |  |  |  |  |  |  |  |  |
| Cu | Children | 8.160E-06 | 2.281E-10 | 2.285E-14 | 8.161E-06 |  |  | 3.998E-08 | | 2.545E-12 | 2.151E-10 | - |  |
|  | Adult | 8.743E-07 | 1.286E-09 | 3.489E-15 | 8.756E-07 |  |  |  |  |  |  |  |  |
| Se | Children | 5.333E-09 | 1.490E-13 | 1.493E-17 | 5.334E-09 |  |  | 3.265E-12 | | 2.079E-16 | 1.7567E-14 | - |  |
|  | Adult | 5.714E-10 | 8.403E-14 | 2.280E-18 | 5.715E-10 |  |  |  |  |  |  |  |  |
| Cd | Children | 2.933E-07 | 8.196E-12 | 8.213E-16 | 2.934E-07 |  |  | 1.796E-11 | | 1.144E-15 | 9.663E-14 | 6.862E-12 |  |
|  | Adult | 3.142E-08 | 4.622E-12 | 1.254E-16 | 3.143E-08 |  |  |  |  |  |  |  |  |
| Pb | Children | 6.068E-06 | 1.695E-10 | 1.593E-14 | 6.069E-06 |  |  | 2.601E-09 | | 1.656E-13 | 1.399E-11 | 2.223E-11 |  |
|  | Adult | 6.502E-07 | 9.562E-11 | 2.595E-15 | 6.503E-07 |  |  |  |  |  |  |  |  |

1. **Phlogopite**

|  | | Non-Carcinogenic Health Risk | | | |  | Carcinogenic Health Risk | | | |
| --- | --- | --- | --- | --- | --- | --- | --- | --- | --- | --- |
|  |  | HQ_ing_ | HQ_inh_ | HQ_der_ | HI=∑HQ |  | LADD_ing_ | LADD_inh_ | LADD_der_ | ILCR |
| Cr | Children | 4.974E-04 | 1.390E-08 | 1.393E-12 | 4.974E-04 |  | 1.827E-08 | 1.163E-12 | 9.829E-11 | 1.837E-08 |
|  | Adult | 5.329E-05 | 7.837E-09 | 2.127E-13 | 5.329E-05 |  |  |  |  |  |
| As | Children | 1.395E-04 | 3.896E-09 | 3.905E-13 | 1.395E-04 |  | 5.123E-09 | 3.262E-13 | 2.756E-11 | 5.152E-09 |
|  | Adult | 1.494E-05 | 2.197E-09 | 5.962E-14 | 1.495E-05 |  |  |  |  |  |
| Ni | Children | 5.752E-06 | 1.607E-10 | 1.611E-14 | 5.752E-06 |  | 1.409E-08 | 8.969E-13 | 7.579E-11 | 1.416E-08 |
|  | Adult | 6.162E-07 | 9.063E-11 | 2.459E-15 | 6.164E-07 |  |  |  |  |  |
| Cu | Children | 6.850E-06 | 1.914E-10 | 1.918E-14 | 6.852E-06 |  | 3.356E-08 | 2.137E-12 | 1.805E-10 | - |
|  | Adult | 7.341E-07 | 1.079E-09 | 2.928E-15 | 7.351E-07 |  |  |  |  |  |
| Se | Children | 1.067E-08 | 2.980E-13 | 2.987E-17 | 1.067E-08 |  | 6.531E-12 | 4.158E-16 | 3.514E-14 | - |
|  | Adult | 1.143E-09 | 1.681E-13 | 4.56E-18 | 1.143E-09 |  |  |  |  |  |
| Cd | Children | 5.066E-07 | 1.415E-11 | 1.418E-15 | 5.067E-07 |  | 3.102E-11 | 1.975E-15 | 1.669E-13 | 3.119E-11 |
|  | Adult | 5.428E-08 | 7.983E-12 | 2.166E-16 | 5.490E-08 |  |  |  |  |  |
| Pb | Children | 8.823E-06 | 2.465E-10 | 2.316E-14 | 8.823E-06 |  | 3.781E-09 | 2.408E-13 | 2.034E-11 | 3.802E-09 |
|  | Adult | 9.453E-07 | 1.390E-10 | 3.772E-15 | 9.460E-07 |  |  |  |  |  |
